# Miscellaneous Abstracts

**Published:** 1894-09

**Authors:** Wm. Keiller

**Affiliations:** M. D., F. R. C. S., Ed., Professor of Anatomy University of Texas; late Physician for Diseases of Women, Edinburgh Providence Dispensary


### MISCELLANEOUS ABSTRACTS.

BY WM. KEILLER, F. R. C. S., ED.,
Professor of Anatomy University of Texas; late Physician for Diseases of Women, Edinburgh Providence Dispensary.

SURGERY.—Surgery of the Spinal Cord and its Appendages.—*Thorburn*, *British Medical Journal*, June 23, 1894.

1. INJURIES.—In penetrating wounds of the spinal cord, death is usually due to spreading septic meningitis. The discharge of cerebro-spinal fluid gives no trouble. The proper treatment therefore of penetrating wounds (compound fracture) is to clear out the wound, leaving it open to drain freely. The cauda equina may be sutured successfully, but there is no hope in suture of the spinal cord.

*Simple Fractures.*—1st. Fractured spinous process alone, is rare, and is seldom accompanied by compression of the cord. If there be compression, the depressed spine should be removed. 2d. Fractures of laminæ may be due to direct or indirect violence, and are difficult to diagnose, the only reliable sign being lateral mobility of the corresponding spinous process. Here laminectomy is clearly indicated if there be symptoms of compression. 3d. Fracture dislocation of the vertebral bodies. Dislocation may or may not be accompanied by fracture. The commonest form is a luxation. It may be unilateral or complete. The bones may recoil again to their proper position. Compression of the cord is seldom permanent, but the temporary crush may injure beyond recovery. Rupture of vessels without crush of cord may take place, causing a gradually ascending paralysis from pressure of blood clot. In this last case exposure of cord at site of injury, combined with aspiration lower down, may remove pressure and relieve symptoms; in all other cases of fracture, dislocating the vertebral bodies, operation is contraindicated. Below the level of the first lumbar vertebra, however, operation may give relief.

2. CARIES.—Caries may effect the vertebral bodies or the arches, the former far more commonly. In the cases (a) paraplegia is seldom caused by mere kyphosis. (b). Sudden paraplegia may be caused by fracture due to caries. Extension and fixation of the spine is the most suitable treatment. (c). Rare causes of paraplegia are bursting of abscesses into the canal, hemorrhage into the canal, or displacement of a sequestrum. (d). The usual cause of paralysis in vertebral caries is granulation tissue, the pressure being accompanied by irritative nontubercular pachymeningitis. (e). A few cases are due to tubercular periarteritis of the vessels of the cord, the diseased process spreading from the bony focus.

*Prognosis.*—Except in class (e) recovery (seldom perfect, however,) occurs after prolonged rest (two cases of recovery after eighteen months' total paralysis recorded). Relapses are common both in these cases and in those subjected to operation.

*Indications for Operation.*—Recovery usually following rest, operation is only indicated:

1. Where symptoms steadily increase in spite of the most favorable conditions.

2. Where symptoms threaten life; e. g., secondary chest trouble, cystitis.

3. By persistency of the symptoms in spite of complete and prolonged rest.

4. In caries of the arches. Here it is possible to remove the whole tuberculous tissue.

5. Where pain is excessive.

6. Other things being equal, children yield the best results after operation.

*Contraindications.*—Active tuberculosis elsewhere, pyrexia unless it be due to cystitis, fracture of various vertebræ.

*Mortality due to Operation.*—Probably 17 to 20 per cent., the most common cause of death being shock.

*Results.*—Often great improvement at first, but relapses are very common. The operation, of course, is laminectomy with removal of all diseased tissue that can be reached.

3. SPINA BIFIDA OCCULTA.—A rare disease, described by Virchow in 1875. As a result of a small slit in a vertebral arch, there develops slowly a hypertrophic process accompanied by pressure on the spinal cord (cauda equina usually), external swelling, and usually a local excess of hair. The symptoms are late occurring and slowly developing trophic lesions (e. g., perforating ulcer of the foot), anæsthetic or (rarely) painful areas, paresis. The cases seem suitable for operation, and one operation with excellent result is recorded.

4. Other diseases as tumors, etc., were mentioned briefly and are to be treated, each, on its own merits.

---

CASTRATION FOR HYPERTROPHY OF PHE PROSTATE.—*British Medical Jonrnal*, June 23, 1894. White, of Philadelphia, calls attention to the accumulating evidence in favor of castration as a remedy for prostatic hypertrophy. In 1892, arguing from the relationship between the prostate and uterus, and prostatic hypertrophy and fibromyomata, he had experiments carried out on dogs, which showed that castration caused decrease in the size of the prostate, and then suggested the operation. Since then Ramm, of Christiana, has operated twice; Powell reported a case in point; Haynes, of Los Angeles, has operated three times; Fremont Smith once, and White once, all with perfect relief of symptoms (frequent micturition, necessity of catheter, cystitis, blood in urine, etc). In one other case division of the vasa deferentia gave no result. He thinks the claim of the operation to more extended trial is fully established.

---

THE PITUITARY BODY.—*British Medical Journal*, June 23, 1894. Evidence, experimental and surgical, is accumulating that the pituitary body has a trophic influence on the brain. The researches of Dr. Andriezen, and of Vassali and Sacchi, show that its destruction cause (a) a fall of temperature to subnormal; (b) anorexia, listlessness, progressive emaciation; (c) fibrille twitchings, then cramps and spasms of the muscles; (d) attacks of dyspnœa; (e) final death.

Woolcombe relates a case of psammoma of the pituitary body in a girl eleven years old, who had all the above symptoms well marked except c and a. In addition she had headache and symptoms due to pressure on the optic commissure and third nerves. Regarding her symptoms, he notes, 1st, the depression and apathy were such as could not be produced by a tumor of the same size (hen's egg) in any other part of the brain. 2nd. The early and rapidly developing muscular weakness was greater than a similar tumor anywhere else could produce. 3rd. Subnormal temperature was a valuable sign, and this and the other symptoms seem to connect the function of the pituitary body with that of the thyroid gland. She died in five months from the appearance of her first symptoms.

---

THE ANTITOXIN TREATMENT OF DIPHTHERIA.—*British Medical Journal*, June 23, 1894. Fizzoni and Cattani have recently had excellent results in the treatment of diphtheria by blood serum from immunized goats. 220 children were treated.

Six cases, admitted on first day, all recovered; sixty-six, on second day, with two deaths, and in these two tracheotomy was necessary; of twenty-nine cases, admitted on the third day, four died; of thirty-nine, admitted on the fourth day, nine died; of twenty-three, treated on the fifth day, ten died. Of the whole 220 cases, of whom sixty-seven required tracheotomy before treatment was commenced, fifty-two died, and 168 recovered. The treatment is being tried in Paris with satisfactory results. Full details will soon be published, and it is hoped the method will be rapidly put to a general test. In most cases only a single injection was made.

---

SMALL-POX DEATH RATE and vaccination of fifty small-pox patients admitted to the North Brierley Joint Hospital, England, during the eighteen months ending December, 1893, forty-four had been vaccinated at some time during their lives, and all recovered; six had never been vaccinated, and of them three (50%) died! When will the anti-vaccination prejudice be rooted out? Truly, some British subjects, and others, are pig-headed.

Small-pox and vaccination in 1893, in Birmingham, England. Dr. Hill's report. *British Medical Journal*, July 7, 1894:

| AGES. | VACCINATED. | | |
|---|---|---|---|
| | Cases. | Deaths. | Case Mortality per cent. |
| Under 1 year................ | 0 | 0 | — |
| Between 1 and 5 years........ | 6 | 0 | — |
| Between 5 and 15 years....... | 101 | 0 | 0 |
| Between 15 and 25 years...... | 358 | 6 | 2 |
| Between 25 and 45 years...... | 316 | 22 | 7 |
| Between 45 and 65 years...... | 58 | 8 | 14 |
| At 65 years and upwards ..... | 8 | 2 | — |
| AGES. | UNVACCINATED. | | |
| Under 1 year................ | 14 | 6 | 43 |
| Between 1 and 5 years........ | 21 | 12 | 57 |
| Between 5 and 15 years....... | 34 | 2 | 6 |
| Between 15 and 25 years...... | 18 | 4 | 22 |
| Between 25 and 45 years...... | 16 | 7 | 44 |
| Between 45 and 65 years...... | 1 | 0 | — |
| At 65 and upwards........... | 1 | 1 | — |

